# SHARE: Sustain Health development in Africa through Responsible Education

**Published:** 2016

Founded nine years ago by Prof Jean Marco (France) and Dr Francois Bourlon (Monaco), who has been largely instrumental in driving it, SHARE is a non-profit organisation that provides essential training to African cath lab staff by linking them with experienced teams in Europe, the USA and Asia Pacific. It’s aimed at those African cardiologists who wish to build a local diagnostic and interventional practice and need to develop their skills.

‘Africa has 1.1 billion people in 54 countries, only 20 of which have a cath lab’, said Dr Bourlon. ‘We therefore saw a need for a training organisation, which is funded by the private sector and industry partners.’

SHARE’s first initiative was the establishment of a medicosurgical centre in Nouakchott, Mauritania, North Africa. ‘We started from nothing, which makes it a significant achievement. The centre comprises one cath lab, two operating theatres, one recovery room, one sterilisation unit and one in-patient unit. With the assistance of the Europa Organisation, we have trained four cardiologists and four allied health professionals, who committed to return to Mauritania once their training was finished and submit written activity reports every three months. The unit is now autonomous, undertaking angiography, percutaneous interventions and cardiac surgery. It has even organised its own congress.’

Now SHARE is facilitating the training of an interventional cardiologist in mitral valvuloplasty. He is currently based in Morocco, further to a year of training in Italy. ‘We’re also developing training material for nurses and allied professionals in the form of short educational films that will be made available on YouTube’, said Dr Bourlon.

With its Mauritanian initiative successfully completed, SHARE has turned its attention to Mali in West Africa with a view to duplicating the Mauritanian experience. ‘There is currently no such facility in the capital, Bamako, so we are looking to design and build a cath lab there. The initial steps are under way, including one cardiologist currently being trained in Monaco.’

SHARE’s vision for the future is to consolidate its actions in Mauritania and Mali, and extend this vision to other African countries. ‘We’re looking to find new training centres in both Europe and Africa and facilitate the participation of trained personnel in educational activities. We wish to create a community of learners who are united in a shared experience. We accept that progress is slow, but we keep moving forward step by step’, concluded Dr Bourlon.

